# Th17-related mammary immunity, but not a high systemic Th1 immune response is associated with protection against *E. coli* mastitis

**DOI:** 10.1038/s41541-020-00258-4

**Published:** 2020-11-24

**Authors:** Nathan Cebron, Sarah Maman, Sarah Walachowski, Blandine Gausserès, Patricia Cunha, Pascal Rainard, Gilles Foucras

**Affiliations:** 1 IHAP, Université de Toulouse, ENVT, INRAE, 31076 Toulouse, France; 2 SIGENAE, GenPhySE, Université de Toulouse, INRAE, INPT, ENVT, 31326 Castanet Tolosan, France; 3 ISP, INRAE, Université de Tours, 37380 Tours, France

**Keywords:** Vaccines, Bacterial infection

## Abstract

Vaccination against bovine mastitis lags behind despite high demand from the dairy industry and margin for efficacy improvement. We previously compared two immunization protocols against *E. coli* using either only the intramuscular route or a combination of intramuscular and mammary ductal routes, also known as ‘prime and pull’ strategy. A homologous mammary challenge during the memory phase showed that immunization favorably modified the mastitis course, notably in locally immunized cows in comparison to intramuscular and control adjuvant-only groups. Here, we performed whole-blood profiling through RNA-seq transcriptome and plasma cytokine 15-plex analyses at time points of the *E. coli* mastitis that showed significant clinical and laboratory differences among the groups. Diminished production of inflammatory cytokines and increased IFNγ were detected in the blood of immunized cows, where a T lymphocyte activation profile was evidenced at 12-h post infection. Acute phase neutropenia was less severe in these cows, and pathways related to neutrophil diapedesis and monocyte activation were also present. Furthermore, three intramammary-immunized cows showing faster healing and shorter mastitis duration had gene profiles that differed from their counterparts, but without any clue for the mastitis susceptibility difference. Inasmuch, when gene expression of CD4 T cells was assessed in mammary tissue, enrichment of IL-17-associated pathways was identified in the quarters of intramammary-immunized cows not only after challenge but also in the control quarters that were not infected. These findings indicate that local immunization mobilizes protective mechanisms that rely on the settlement of type 3 immunity-related CD4 T cells prior to infection.

## Introduction

Mastitis is a major disease of dairy cows^1^, which develops upon infection of the mammary gland by various bacterial species. Clinical cases are mainly due to *Escherichia coli* and *Streptococcus uberis*, while *Staphylococcus aureus* is more frequently isolated from chronic subclinical mastitis. Infection can easily be detected due to leukocyte recruitment in mammary tissue, and ultimately in milk, leading to an increased milk somatic cell count (SCC), an indicator widely implemented in dairy control programmes^2^.

Mastitis prevention and cure heavily rely on antibiotics, so that mastitis is the main reason for their common usage in dairy cattle^3^. Alternatives to antibiotic treatment are needed to reduce their use and the spread of antibiotic resistance^4^. Development of strategies to increase mammary defences is a major possibility, and among immune-based approaches, vaccination would be a good path to follow^5^. Unfortunately, marketed vaccines based on bacterins, such as the *E. coli* J5, suffer a certain number of limitations that lessen their generalized utilization in dairy herds. These vaccines have shown a low reduction of clinical mastitis incidence upon natural exposure, and they mainly reduce the severity of mastitis and milk losses^6,7^. Since new developments are needed to overcome the low efficacy of available commercial vaccines, the slow progress is partly due to the lack of knowledge about the way vaccines operate in the mammary gland and about the exact protective mechanisms against infection.

In effect, the local immune response relies essentially on early and efficient recruitment of neutrophil granulocytes to control bacterial infections. Antibodies in the IgG1 and G2 isotypes, and to a lesser extent IgA since their concentration is lower in ruminants compared to other mammals, do not seem to play a major role although they are generally induced by current vaccines.

Vaccine delivery via mucosal routes is an interesting alternative to parenteral administration^8^. Local immunization through respiratory, digestive, and genital mucosal surfaces has been shown to induce local immunity associated with tissue-resident CD4 and CD8 memory T cells^9–11^. The mammary gland does not have a mucosal surface strictly speaking, although it is sometimes affiliated to the mucosal common immune system. Surprisingly, the induction of a robust immune response against mammary pathogens through intramammary (IMM) immunization has rarely been reported in dairy ruminants. Pioneering work by Lascelles^12^ used living staphylococci in dairy ewes, but the main limitation of these studies is that no information is available on the mammary status and ensuing inflammation before injection of the challenge inoculum. The presence of high SCC at the time of experimental infection may have prevented the proliferation of the bacterial inoculum and thus the development of mastitis. More recently, immunization locally with killed *Streptococcus uberis* had favorable effects on the reduction of mastitis frequency on the next lactation in dairy cows^13^. Local injection with a low number of dead *E. coli* bacteria also had some protective effects, although it is doubtful if immunity was really present at the time of challenge due to the low severity of the induced mastitis in the control group^14^.

In a vaccination trial against bovine *E. coli* mastitis previously reported by us^15^, we used a local administration of *E. coli* crude antigens after a primary intramuscular (IM) immunization, also known as ‘prime and pull’ strategy. Compared to IM route alone, we showed that the booster injection via the ductal route improved the immunization protocol efficacy. The antibody response did not seem to play a significant role in the immune defences in this case, and the development of a cell-associated immunity with interferon-γ (IFNγ) production in the milk of immunized cows was evident. Indeed, the cell-mediated response may be essential to improve the protective response against *E. coli* mastitis.

To improve our knowledge about vaccine-based cellular immunity against *E. coli* infection, we performed a whole-blood transcriptome analysis on the samples collected during the mastitis trial, and also the immunization phase of the study previously described^15^. By combining high-throughput assessment of whole-blood transcriptome and cytokine protein expression, we describe here the systemic response during *E. coli* mastitis. Furthermore, we provide evidence that the settlement of interleukin-17 (IL-17)-producing T helper cells in the mammary tissue improves immunity to *E. coli* mammary infections.

## Results

### Clinical and biological differences among experimental groups upon *E. coli* mammary challenge

**Fig. 1:**
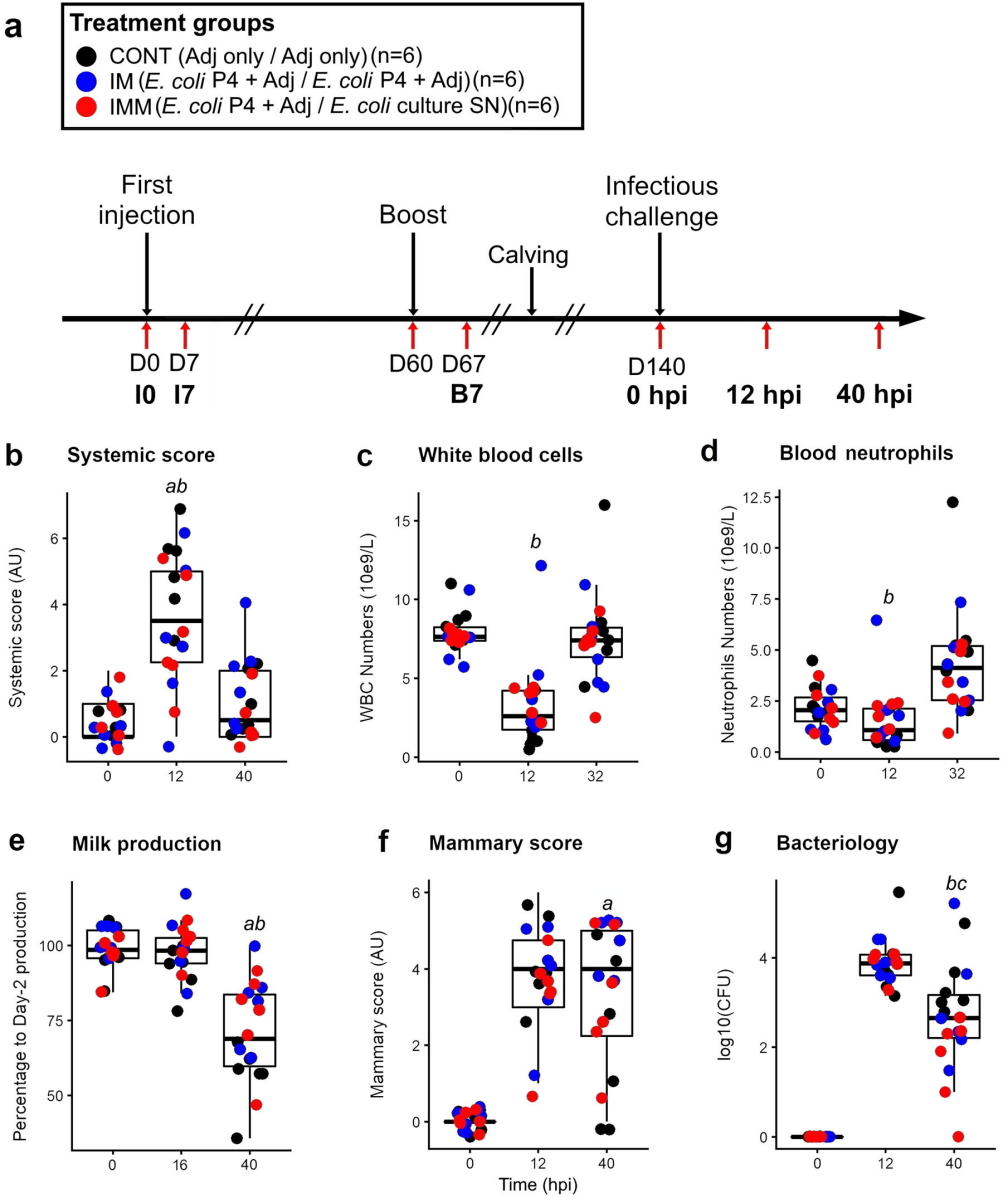
Clinical, hematological, and mammary response of the cows according to their immunization group at three informative time points of the *E. coli* mastitis course. **a** Experimental scheme with immunization and experimental challenge phases. Systemic (**b**) and mammary (**f**) clinical scores were established as composite values of several signs recorded by observers blind to the treatment. Milk production expressed as the relative percentage of milk produced at each time compared to the average production before inoculation (**e**). Milk bacteriology (**g**) expressed as the log 10 value of colony-forming units per mL of milk, white blood cells (**c**) and neutrophils (**d**) counts are presented at the same times. Each dot corresponds to an individual cow with colors indicating the treatment group and the box plot showing the mean and standard deviation at each time point. Letter indicate a difference (Kruskal–wallis, *p* < 0.05) between groups (**a**: CONT vs IM; **b**: CONT vs IMM; **c**: IM vs IMM).

The mastitis trial contained two different immunization treatment groups (IM or IMM, *n* = 6 per group), and a control (CONT, *n* = 6) group that only received the same adjuvant intramuscularly at the indicated dates (Fig. 1a). The ensuing results have been previously described in more details over a course of 10 days^15^. Here, we summed up the main clinical and biological differences observed during the challenge. Indeed, in order to look for mechanisms and the specific effects of each immunization scheme, three time points (0, 12, and 40 hpi) were selected as the most discriminant among the groups, where significant differences were evidenced (Kruskal–Wallis, *p* < 0.05), and corresponding to the acute (12 hpi) and the late (40 hpi) phases of the *E. coli* mastitis. Several criteria are considered: the systemic clinical score (Fig. 1b), white blood cell (Fig. 1c**)**, and neutrophil (Fig. 1d) counts; milk production (Fig. 1e); the mammary clinical score (Fig. 1f), and milk bacteriological load (Fig. 1g).

In addition, blood samples collected at day 0 (I0), and 7 days after the IM immunization (I7) and the booster injections (B7) were included in the analysis. In total, 107 samples from six dates and six animals in each of the three groups were prepared for transcriptome analysis; one sample (CONT, 12 hpi) was missing due to the low amount of RNA recovered after extraction. The whole-blood transcriptome was established using RNA sequencing with an average value of 10.3 million (R1 + R2) reads for each sample (Supplementary Table 1). The dataset obtained with FeaturesCount after STAR alignment comprised 24,617 bovine genes. At the acute phase of *E. coli* mastitis (12 hpi), the number of reads was significantly lower than at the other time points (Kruskal–Wallis, *p* < 0.05), except in the IMM group (Supplementary Table 1). One sample (IM, B7) was removed from the dataset due to an outlier profile in the principal component analysis (PCA). In total, 106 samples were available for the statistical analysis.

### Whole-blood transcriptome through RNA-seq identifies the main biological functions altered by *E. coli* mastitis

**Fig. 2:**
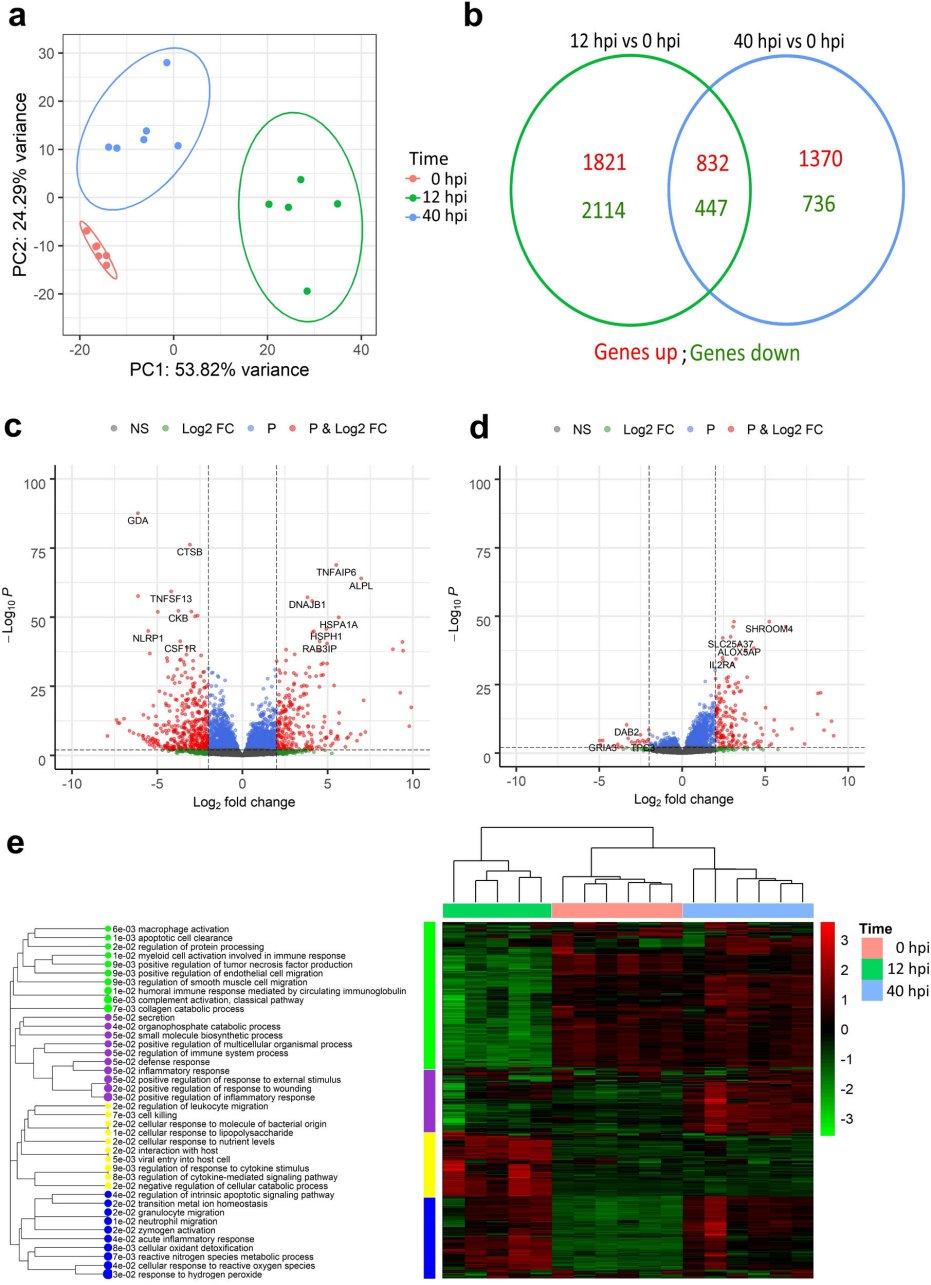
Whole-blood transcriptomic response of control cows during the course of an *E. coli* mastitis. Differentially expressed genes (DEGs) between times were assessed using Deseq2. **a** A principal component analysis on the top 500 most differentially expressed genes of control cows at 0, 12, and 40 h post infection (hpi) shows that the expression profile of blood cells changed according to the time during the *E. coli* mammary infection. A Venn diagram presents the number of DEGs between time points, and the number of genes in common between the two comparisons (between 12 and 0 hpi and between 40 and 0 hpi) (**b**). Volcano plots were used for determining DEGs in the CONT group between 12 and 0 hpi (**c**), and 40 and 0 hpi (**d**). Thresholds were arbitrarily established for a *q* value < 0.01 and a log2FoldChange > 2 and genes above these thresholds are depicted in red dots. **e** A Heatmap of the top 500 DEGs during infection in control group is presented using Euclidian distribution analysis. Results of the functional analysis with ToppFun is presented as a dendrogram showing the relationship among biological functions and blocks of commonly regulated genes.

As a blood transcriptome related to bovine *E. coli* mastitis has never been reported before, we first investigated changes in gene expression in the CONT group. A PCA showed that the expression profile varied according to the time, with a clear separation between 0 and 12 hpi (PCA axis 1: 53.8%), and between 0 and 40 hpi (PCA axis 2: 24.3%) (Fig. 2a). Differentially expressed genes (DEGs) were identified with DESeq2 at these two time points in a comparison with the pre-inoculation time point. The numbers of DEGs were 5214 (with 2653 over- and 2561 under-expressed genes), and 3385 genes (2202 over and 1183 under), at 12 and 40 hpi, respectively. DEG lists were mostly different at the two times, as a minority of genes was common between the two sets as shown in a Venn diagram (with 832 over and 447 downregulated DEGs) (Fig. 2b). Volcano plots showed determining DEGs for the 12 hpi (Fig. 2c) and 40 hpi (Fig. 2d) comparisons, and the lists of the top 10 over- or under-expressed DEGs are available in Supplementary Table 2 for 12 and 40 hpi time points. To further depict the response and identify blocks of commonly regulated genes and functions, a heatmap was prepared (Fig. 2e), with samples clustering according to the time. The top 500 most DEGs were divided into four modules that typified individual time points. The function modules were determined with ToppFun software and are presented with a dendrogram to evaluate correlations among functions. At 12 hpi, *Cellular response to lipopolysaccharide and Regulation of cytokine mediated signaling pathway* were the most activated functions, while *Humoral response* and *Macrophage activation* characterized the late phase at 40 hpi. *Neutrophil migration* and *Cellular response to reactive nitrogen species* were significantly detected at both times. Plasma concentrations of IL-6, tumor necrosis factor-α (TNFα), IL-10 cytokines, and CCL2, CCL4, CXCL10 chemokines were significantly increased at 12 hpi (Kruskal–Wallis, *p* < 0.01), and returned to initial values at 40 hpi; only IL-1RA plasma concentration was increased at both 12 and 40 hpi (Supplementary Fig. 1).

### Comparison with the transcriptome profiles of IM- and IMM-immunized cows

**Fig. 3:**
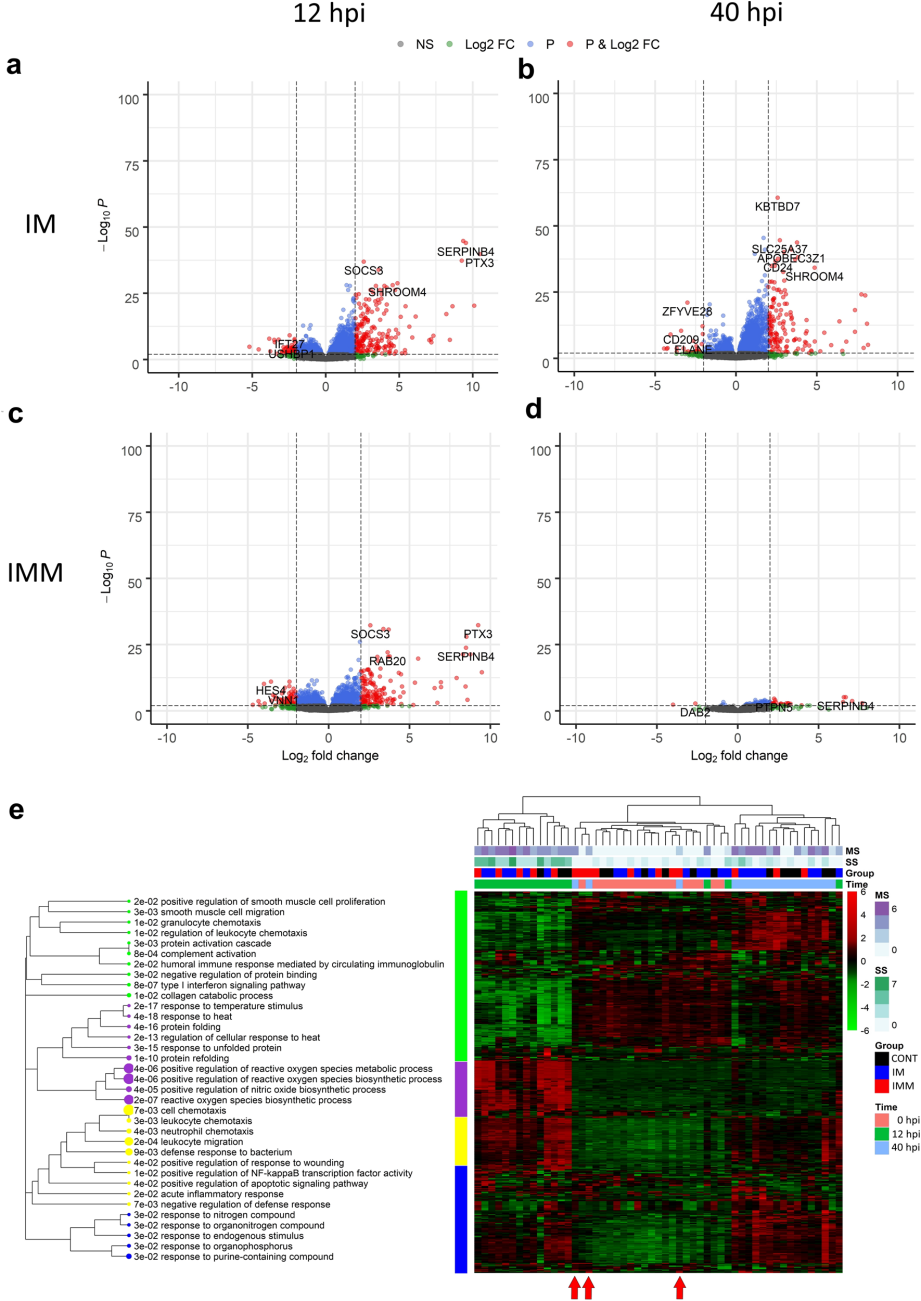
Whole-blood transcriptomic analysis of the response of immunized cows during the *E. coli* mastitis course. DEGs at two time points (12 and 40 hpi) in comparison with 0 hpi were identified with Deseq2. Volcano plots comparing gene expression differences between 0 and 12 hpi (**a**, **c**), and at 0 and 40 hpi (**b**, **d**) are presented for the intramuscular (IM) and the intramammary (IMM) group, respectively. Red dots correspond to genes with a *q* value < 0.01 and a Abslog2FoldChange > 2. **e** Heatmap of the top 500 DEG in control and immunized groups during the course of infection is presented. Functional analysis was performed with ToppFun and a dendrogram shows the relationship among biological functions of commonly deregulated genes according to the mammary and systemic scores, the time, and the treatment group. MS mammary score, SS systemic clinical score. Red arrows indicate IMM cows with 0 and 40 hpi profiles that were undistinguishable (further designed as cured cows).

In order to compare the blood transcriptomes among the groups, we first looked for DEGs in the two immunized groups, independently. After DESeq2 analysis, DEGs were identified at 12 and 40 hpi in both groups. The lists showing the top 10 genes with up and down expression in IM and IMM groups are provided in Supplementary Tables 3 and 4. The DEG numbers were not different between the groups at 12 hpi (Fig. 3a, c), but it was much lower in the IMM group at 40 hpi (Fig. 3b, d). Venn diagrams are presented in Supplementary Fig. 2 to determine the number of commonly deregulated genes. Interestingly, the number of DEGs was lower in the immunized cows compared to the controls at 12 hpi, and the difference was even larger for the IMM group at 40 hpi, where DEG number represented only 10% of the numbers obtained in the IM and CONT groups, supporting the idea that systemic healing was earlier in this group. Altogether, samples clustered essentially according to the Time in a global heatmap (Fig. 3e), except three samples from IMM cows at 40 hpi that grouped together with the samples collected before inoculation. Bacterial excretion in these cows was the lowest after this time point (<100 bacteria/mL) and their mammary infection was actually cleared after 48 h, and twice faster than other cows. Indeed, expression profiles of these three IMM cows cannot be distinguished between 0 and 40 hpi, indicating that gene expression almost returned to the pre-infection state in these cows. They are further defined as cured cows on the basis of both their lower clinical scores and their gene expression profiles. The top 500 DEGs divided into four modules determined with ToppFun software and were essentially related to immune functions (Fig. 3e). At 12 hpi, activated functions were *Defense response to bacterium*, and *Neutrophil chemotaxis*, as shown before for the CONT group. At 40 hpi, *Type I interferon signaling* and *Humoral immune response* were significantly activated in the IM group, which showed the slowest recovery rate. The profiles were identical at time 0 hpi among the groups, indicating the absence of any ongoing immune activation or physiological difference among the groups. Plasma cytokine dosage showed that the production of pro-inflammatory cytokines IL-1β, IL-6, and TNFα, but also CCL2 and CCL4 chemokines was significantly lower in the immunized compared to the CONT cows at the acute phase (12 hpi), indicating a lower inflammatory response upon infection in the IM and IMM groups (Supplementary Fig. 3). Furthermore, IFNγ and CXCL10 concentrations were higher in the IM group at 40 hpi, in agreement with the activation of interferon-related pathways in this group during the late phase of the response (Fig. 3e).

To identify the biological functions that correlated with the systemic and mammary scores as presented above in Fig. 1b, f, we used weighted correlation network analysis (WGCNA) on this set of gene expression data. Twelve modules significantly correlated with the mammary and/or systemic scores. Notably, a T helper type 1 cell-related module was activated at 12 hpi in the immunized groups, indicating that immunization protocols through either IM or IMM routes changed the course of the immune response during mammary infection (Supplementary Fig. 4). These results also suggest that immunization promoted a T cell response that was mobilized in blood early during the mastitis course.

### Systemic and local routes of *E. coli* immunization elicit different gene expression profiles

**Fig. 4:**
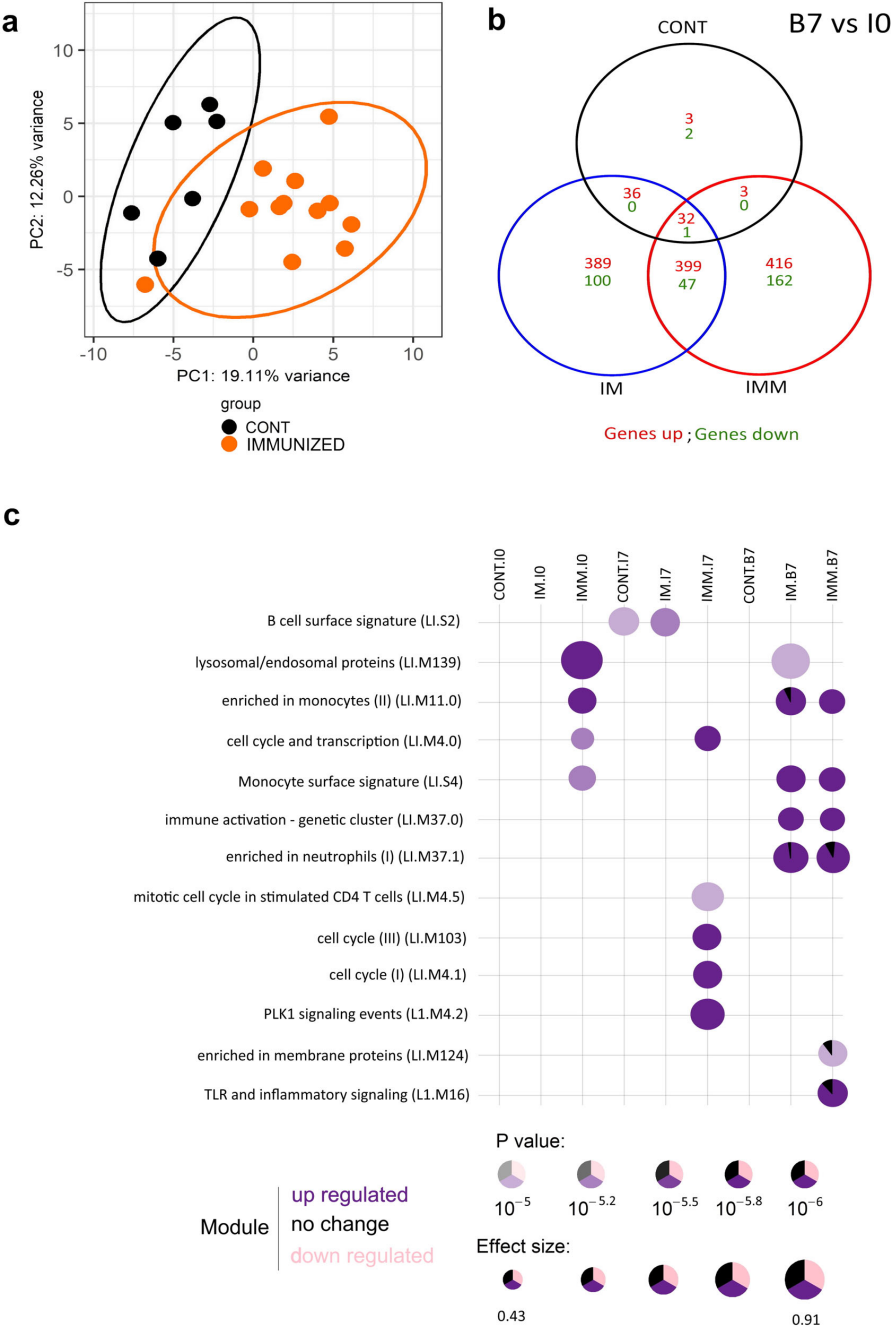
Whole-blood transcriptomic response to immunization in control and immunized groups. **a** A principal component analysis (PCA) on the top 500 most DEG of CONT (black) and immunized IM + IMM (orange) cows after the first injection (I7) shows that the expression profile of blood cells changes upon systemic immunization. **b** Venn diagram of identified genes in control, intramuscular and intramammary groups at 7 days after booster injection (B7) compared to I0 (before immunization). **c** A modular analysis on B7 gene expression shows the significant difference (*p* value < 10^−5^) of upregulated biological functions between CONT, IM, and IMM groups.

Since immunization changed the susceptibility to *E. coli* mastitis, we investigated DEGs after the first intramuscular and booster injections to decipher the immune-related functions mobilized by each kind of immunization scheme. The comparison of transcriptome data did not reveal any difference among the groups before immunization (I0). Analysis of the primary response at 7 days post-IM injection (I7) indicated that 600 genes were DE in the *E. coli*/adjuvant (*n* = 12 cows) compared to the adjuvant alone condition (*n* = 6 cows) (Fig. 4a). DEGs were related to immune functions with *Secretion, Myeloid leukocyte activation, and Immune effector process* (Supplementary Table 5). After the booster injection (B7), through either the IM or the mammary ductal (IMM) routes, gene expression profiles were significantly different among the groups. Interestingly, the number of DEG was greater in the immunized groups compared to the CONT group, with 389 and 416 genes upregulated in the IM and IMM groups, respectively (Fig. 4b). These results suggest that the presence of *E. coli* antigens and microbe-associated molecular patterns like the lipopolysaccharide (LPS), in addition to the adjuvant which was the same between CONT and IM groups had a strong effect on the secondary response. DEGs in the IM group were related to *Interferon alpha/beta signaling* and *Cytokine signaling in immune system* functions, whereas DEGs in the IMM group were related to *Neutrophil degranulation* and *Chemokine signaling pathway* functions. Venn diagram indicated that 399 upregulated genes were common between IM and IMM groups. These genes were related to *Neutrophil degranulation* and *Innate immune system* functions (Supplementary Table 6). Furthermore, while no module was activated in the CONT group, four modules were commonly upregulated in the immunized groups with *Enrichment in monocytes* and *Immune activation—generic cluster* as the main modules. The module *TLR and inflammatory signaling* was activated only in the IMM group at B7 time point (Fig. 4c). So, the injection of antigens from *E. coli* culture supernate in the mammary gland lumen stimulated a systemic response which is essentially different from that induced by the IM immunization with killed bacteria in adjuvant in previously sensitized cows.

### Blood transcriptome profile in IMM cured cows correlated with early cure

**Fig. 5:**
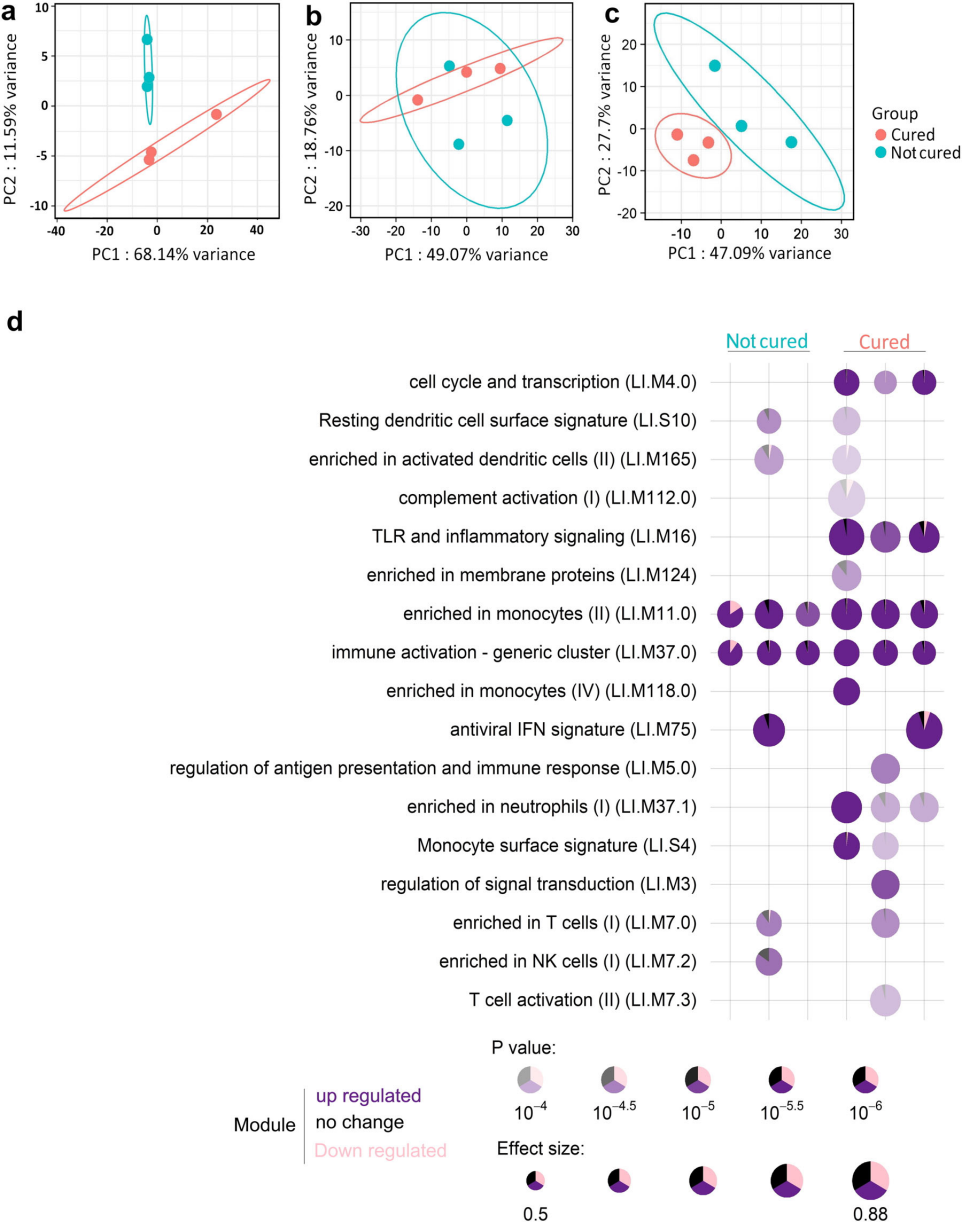
Response upon the mammary booster injection and correlation with the outcome of the *E. coli* mammary challenge. **a** A principal component analysis of gene expression profiles (top 500 most variable genes) for the intramammary-immunized IMM cows at B7. **b** At 12 h post infection and **c** and at 40 h post infection; **d** a modular analysis at B7 shows the difference of upregulated biological functions between cured and not cured cows (*p* value above 10^−4^).

We then paid attention to the comparison between the two IMM subgroups, whose clinical signs and blood transcriptome differed significantly at the late mastitis phase (40 hpi). To define the peculiarities of the cured cows, their blood profile was further scrutinized at all the available dates. A PCA was performed on the top 500 most variable genes from the I0 and B7 data (Fig. 5a). At the B7 time point, PCA showed a significant difference between cured and non-cured cows indicating that the response to immunization of these particular cows was different and correlated with early cure. To further this hypothesis, a modular analysis was performed with the tmod R package. Two modules are common among all animals with *Enrichment in monocytes* and *Immune activation—generic cluster* as the main modules. Three modules were further activated in the cured animals and functions of these modules were *Enriched in neutrophils*, *Cell cycle and transcription*, and *TLR and inflammatory signaling* (Fig. 5d). These functions upregulated in the cured subgroup support the idea that the differences of the systemic response are related to neutrophil mobilization, and are probably an indirect consequence and not the true cause of the difference of cure quickness due to immunization.

To look for the genes and immune functions differentially mobilized between cured/not cured IMM cows during the mastitis, a PCA at 12 and 40 hpi was performed with the top 500 most DEGs, showing similar profiles at 12 hpi, but again a marked difference at 40 hpi (Fig. 5b, c). Analysis with DESeq2 identified only 26 genes (21 up and 5 down in the cured group), whereas at 40 hpi, the number of DEGs was 307 genes with 22 genes upregulated in the cured and 285 in the non-cured subgroup, respectively. Biological functions are presented in Supplementary Table 7. Again, the functional differences do not point to a probable cause of early healing in the three cured cows compared to the rest of the group.

In the comparison between IM and IMM groups, the fact that no DEG, nor function, except neutrophil mobilization, lead to a mechanism explaining the better outcome in locally immunized cows, leaves at least two possibilities: (i) these were very transient during the mastitis course and were not identifiable at the chosen time points for analysis or (ii) the functional differences are not present in the blood, because they reside in the mammary tissue in link with the local immunization.

### Th17-polarized CD4 T cells predominate in the mammary tissue of IMM quarters and are activated after the challenge with *E. coli*

**Fig. 6:**
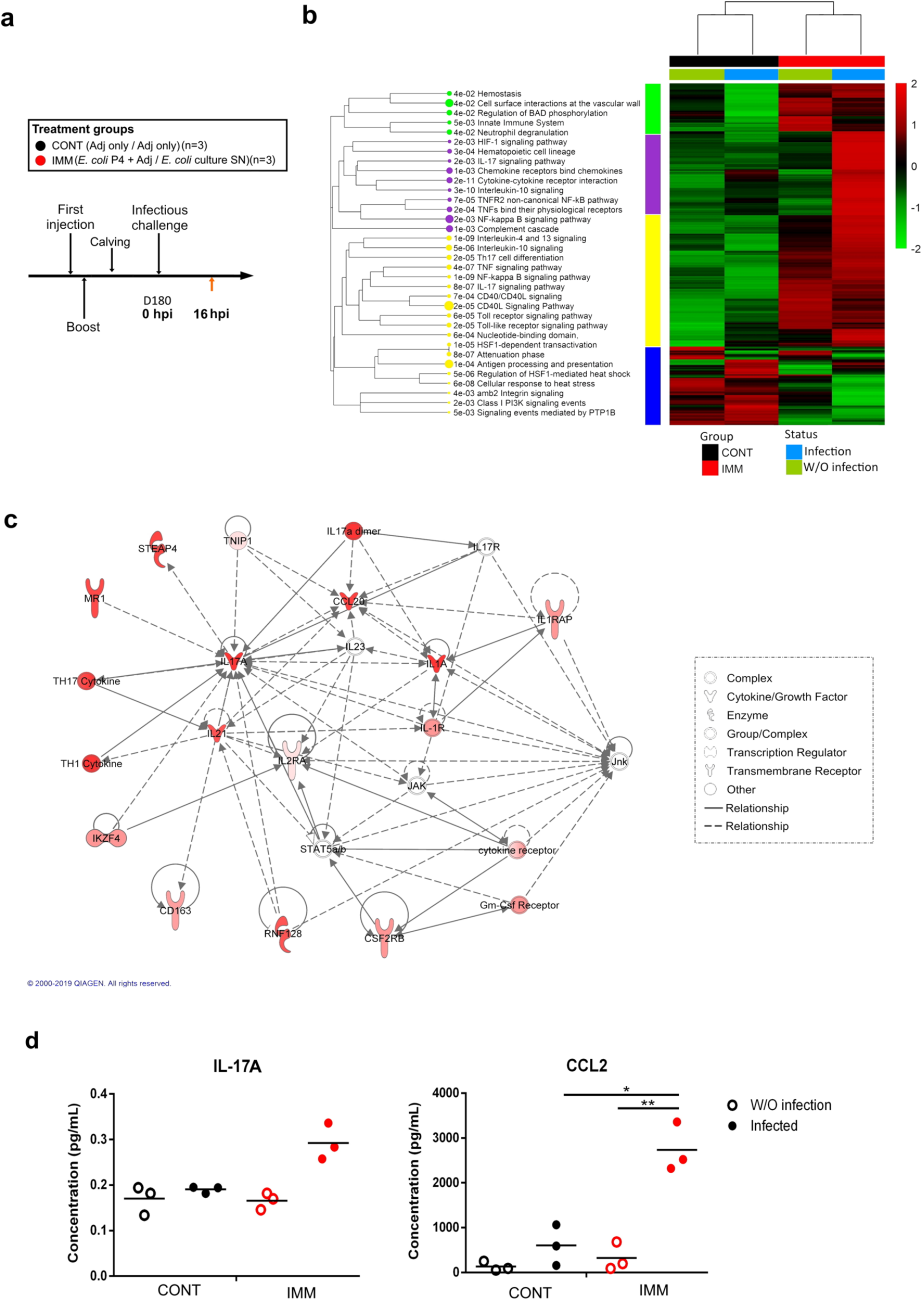
Gene expression profile of CD4 T cells isolated from the mammary tissue of control and challenged quarters from locally immunized and control cows. **a** Experimental scheme with immunization and challenge phases. DEGs were assessed between four conditions corresponding to challenged and control quarters in CONT and IMM cows. **b** A heatmap showing the top 500 DEGs according to the conditions is presented. Functional analysis performed with ToppGene is presented as a dendrogram of functions in each block of correlated genes (*p* value < 0.05). **c** Gene network of the DEGs in CD4 T cells between the infected IMM and infected CONT conditions is presented and shows the relationship with IL-17 cytokine*.* **d** Tissue IL-17A and CCL2 production in non-infected and challenged quarters of CONT and IMM cows was determined on a pre-defined amount of tissue homogenate with a Milliplex assay at 16 hpi. Individual values are shown for *n* = 3 cows per group.

Local immunization has been previously associated with recruitment and establishment of CD4 and CD8 T cells in the targeted tissues. Furthermore, since no difference could be identified in the blood, we made the hypothesis that the mechanisms responsible for the difference of mastitis outcome may develop in the mammary tissue. To examine the phenotype of the T helper cell compartment in link with the mammary immunization, six cows (3 CONT, 3 IMM) were challenged once again using the same conditions (Fig. 6a). CD4 T cells (CD45+ CD4+ cells) were isolated by fluorescence-acquired cell sorting at 16 hpi from mammary tissue upon collagenase treatment. Four different conditions were examined: one challenged and one healthy control quarters from three CONT and three IMM cows. RNA sequencing of pools of purified (purity > 95%) CD4 T cells showed a transcriptional profile where IL-17-related pathways were significantly overrepresented in cells from the IMM cows. Profiles of DEGs were more related to the immunization than to the challenge status of the mammary quarters, suggesting that resident cells of the Th17 phenotype pre-existed the *E. coli* mammary challenge. Analysis of biological functions with ToppFun software is shown on a dendrogram presented in Fig. 6b. The top 500 genes were divided into four modules. Module 1 was related to the IMM group and correlated with *Neutrophil degranulation* and *Regulation of BAD phosphorylation*. Module 3 correlated with *Th17 cell differentiation* and *CD40L signaling pathway*. These modules were expressed in IMM infected and non-infected glands. Module 2 was related to *IL-17 signaling pathway* and *Cytokine-cytokine receptor interaction* and expressed in IMM infected quarters only. Modules related to the infection were mainly correlated with *IL-17 signaling* and *Neutrophil migration*, suggesting that the local Th17 response promoted mobilization of neutrophils, which was protective against *E. coli* infection.

The list of DEG identified with DESeq2 analysis was used to evaluate a difference of pathway activation between the CONT and IMM groups upon infection with Ingenuity Pathway Analysis software. The gene network showed that an IL-17-related network was strongly activated in the IMM group during infection (Fig. 6c).

To confirm the overproduction of IL-17A in immunized quarters, cytokine secretion was measured in milk before *E. coli* inoculation and just before tissue collection at 16 hpi. Despite the low number of animals, IFNγ and IL-17A secretion tend to be higher in the milk of locally immunized cows, while IL-10 secretion was lower (Supplementary Fig. 5), in accordance with the previous report^15^. When cytokines were measured in the tissues of infected quarters, IL-17A, and CCL2, a IL-17-induced cytokine were more abundant in IMM cows (Fig. 6d); a difference of IL-1α was also detected (Supplementary Fig. 6). It is worth noting that milk and tissue concentrations were poorly correlated, except for IFNγ cytokine.

Altogether, these data support the idea that the local booster injection promoted the settlement of basal Th17 immunity in mammary tissue, further amplified by the *E. coli* infection.

## Discussion

Our aim was to decipher the immune mechanisms associated with the local route of immunization which revealed more effective at protecting the mammary gland against infection by *E. coli*. We analyzed blood and milk samples collected on the occasion of our previous challenge study^15^. We provide evidence that the two modes of immunization that were evaluated in parallel mobilized a different immune response. The data strongly suggest that the injection of antigens from *E coli* culture supernate to the local route enabled the establishment in the mammary tissue of Th17-polarized CD4 T cells that were undetectable in non-immunized cows.

We reported previously that the clinical signs of mastitis were less severe in immunized compared to control cows, with faster bacterial clearance and a moderate leukopenia. Nevertheless, there was also a notable difference in response between systemic (IM) and local (IMM) immunization routes, as the inflammatory response persisted longer in the IM group. We suggest now that this was associated with a heightened signaling related to Type I interferon pathway that may be the result of TLR4-Myd88 signaling by LPS released from *E. coli* bacteria in the infected quarters. It could also be the result of a late influx of T cells that was detected in the milk of the IM group after 40 hpi. These cells were circulating in blood at the time of the mammary challenge and were recruited into the inflamed mammary tissue due to the expression of chemokines and endothelial adhesion molecules. Some may have proliferated locally due to antigen recognition or became activated non-specifically by the local production of cytokines. They may thus have contributed to the increase of the inflammatory response during the late phase of the mastitis, while healing was already ongoing in the CONT and IMM groups.

We chose to evaluate blood cell transcriptome by RNA-seq rather than mammary tissue or milk cell gene expression as previously described by us^16,17^ or other groups^18^. Indeed, this approach allowed us to do a kinetics study and to look for mechanisms over time on a tissue whose cell composition is more stable than that of milk during the mastitis course. Indeed, upon infection, recruited cells in milk are essentially neutrophils (>90–95%) as previously shown^15^, whereas these represent no more than a third of the milk cells in healthy glands. Furthermore, RNA quality as defined by RNA integrity number (RIN) assessment is better on blood cells than on milk cells, where many leukocytes are dead or apoptotic/dying cells. If neutrophils represent essential terminal effectors against mammary infection, they do not represent the central type of cells induced by immunization. Conversely, CD4 and CD8 T cells are only a minority in the milk of infected glands, as their number in milk at the time of inoculation is very low (often lower than 10^4^ cells/mL). Later, they are really difficult to isolate from abundant and highly activated neutrophils upon infection with strongly inflammatory Gram-negative bacteria.

We chose to retain six crucial time points, three during the infection course to decipher the protective mechanisms, with 0 hpi corresponding to our time point of reference, and three during immunization with time I0 corresponding to the time point before the first injection of immunization. In the CONT group, the blood transcriptome during the infection phase was informative about the response to an *E. coli* infection, with good clustering of the samples according to the Time. Blood leukocyte gene expression changed quickly and massively upon mammary infection with a large number of deregulated genes. This is partly due to changes in the blood leukocyte composition^15^ but also to changes in cell activation and functions. Indeed, functions related to neutrophils and bacterial recognition are highly activated at the peak of infection, whereas at 40 hpi, these functions are replaced by monocyte/macrophage activation and B cell response. The blood transcriptome actually revealed essential functions that are known to be mobilized or activated during the mastitis course. Blood represents a good compartment to sample to study the immune response to a mammary infection as also shown elsewhere^19^. This may be due to the high blood flow through the lactating mammary gland which is a well perfused, metabolically active organ.

Immunized cows had less significant changes in their blood transcriptome than the controls, in accordance with the systemic changes that were smaller in these cows, as shown in previous reports indicating that immunization with the *E. coli* J5 bacterin diminished clinical signs and associated symptoms like milk production drop, a parameter that is well correlated with the severity of *E. coli* mastitis^20^. Indeed, the number of DEGs at 12 hpi compared to the 0 hpi time point is smaller in the immunized groups compared to the control group with 44% and 57% fewer DEGs for the IM and IMM groups, respectively. On the other hand, at 40 hpi, this decrease is only seen in the IMM group with a 90% decrease of DEGs, confirming the shorter duration of the mastitis in the IMM group in accordance with recorded systemic and mammary clinical signs. This is confirmed by the lower production of inflammatory cytokines (IL-1β, IL-6, and TNFα) and chemokines (CCL2 and CCL4). In addition, high production of IFNy was detected at 40 hpi in the IM group that corresponded to the probable activation of Th1-related CD4 or CD8 T cells that were primed by systemic immunization. This production was lower in IMM cows, maybe because they received only one injection of vaccine in adjuvant or because antigens from *E. coli* culture supernate in the mammary lumen were not able to expand or differentiate the cells to the same magnitude and/or phenotype as did the systemic immunization. As to the IL-17 cytokine, no difference of secretion could be detected in the plasma at any time point^15^.

To look for the pathways correlating with the systemic and/or mammary scores, WGCNA was implemented. It enabled the identification of two different modules that were associated with lymphocytic functions in the IM group and granulocyte degranulation in the IMM group. The indication that T cell activation was weaker in the IMM group compared to the IM group was interesting and led us to investigate CD4 T cells in the mammary tissue, and not only the blood cell compartment.

We provide evidence that IL-17-producing CD4 T cells are enriched in the tissue of bacterial antigen-exposed mammary glands. The antigen repertoire of these cells is not known and whether they are specific to *E. coli* antigens remains to be determined as previously shown for CD8 T cells in another tissue^21^. The role of IL-17 cytokine in immune responses against extracellular bacteria is now well established^22^. This cytokine is known to participate in the local defence by inducing the production of CXCL8 by epithelial and/or stromal cells during infection by fungi and extracellular bacteria, which in turn induces neutrophil influx and antibacterial peptide secretion at the infection site^23^. Its role in bovine mastitis has been highlighted in several reports. In a mastitis model with *E. coli* infection in mice, the IL-17 response in the mammary gland is instrumental in *E. coli* clearance and reduction of mammary inflammation^24^. In dairy cows, despite reports of IL-17 detection in mastitis cases^25,26^, clear evidence was still lacking about its role in reducing mastitis severity.

IL-17-expressing CD4 T cells may be activated by antigen-presenting cells locally or IL-17 expression follows the secretion of IL-23 and activation of T mammary-resident memory (TRM) cells. IL-17 cytokine by acting on epithelial and stromal cells induces CXCL8 secretion and recruitment of neutrophils as shown by the upregulation of this pathway in the modular analysis. Unfortunately, by measuring cell recruitment in milk every 4 h, we were unable to show an influx difference among the groups. The number of cells in milk may not be completely correlated with precocity or intensity of the recruitment in the tissue since neutrophils need to cross the epithelial barrier to reach the mammary lumen and be identifiable in milk. Furthermore, neutrophil bactericidal activity may differ among conditions depending on the cytokine/chemokine pattern related to the type 3 activity they have encountered. Again we were not able to show any difference of opsonophagocytosis and killing activity among the groups using an in vitro assay^15^, suggesting that the antibody response is not central to the mechanisms of bacterial clearance and mastitis healing. Unfortunately, the production of antibacterial peptides was not measured in the secretion of infected quarters to look for other mechanisms related to type 3 immunity.

What determines the phenotype of TRM cells in the mammary gland is another important question to resolve. For priming immunization, we used an adjuvant that is known to promote the development of cellular response in cattle, with a Th1/Th17 profile as previously shown using a model antigen like ovalbumin^27^. When we evaluated IFNγ and IL-17 production using a whole-blood assay with heat-killed bacteria in the same cows, cytokine production in IM and IMM cows was well above that of CONT cows. Although a high degree of variability was seen from cow to cow, IFNγ and IL-17 production increased after the booster injection in the IM group, suggesting that immunization induced circulating CD4 T lymphocytes producing IL-17A and/or IFNγ, contrary to what was seen in the IMM group. Intra-ductal injection of concentrated *E. coli* culture supernate at this step helped to recruit T cells within the tissue. Pro-inflammatory cytokine response induced locally by *E coli*-derived LPS may have increased homing and/or local proliferation of T cells, notably of IL-17-expressing CD4 T cells, as previously described in the lungs^28^ or the intestine^29^ for CD4 and CD8 TRM cells, respectively. Mechanisms of T cell homing within the mammary tissue are probably determinant and merits to be further examined in order to define the best modalities to settle locally more efficient T cell immunity and to improve the immunization protocol efficacy above the results of our proof of concept study.

When studying the response to immunization, blood transcriptome analysis allowed us to identify the presence of differential genes in IM and IMM, while very few DEGs were identified in the CONT group (Fig. 4b). At B7, activation of functions related to monocytes, neutrophils, and T cells were present in the immunized groups. Also, the modules related to inflammatory response and interferon pathway were activated in the IM group, indicating a stronger systemic inflammatory response in the group that received two vaccine injections in adjuvant. PCA showed that the difference of response was already present at B7 time point with upregulated functions remembering what was seen at time 40 hpi, like activation of neutrophil-related functions and TLR signaling. In contrast, profiles were indistinguishable at the peak of infection (12 hpi). This suggests that the cured group of IMM cows had a response at the time of the booster injection that is predictive of more rapid healing upon infection. A genetic control of the response to immunization cannot be excluded at this point to explain this difference.

**Fig. 7:**
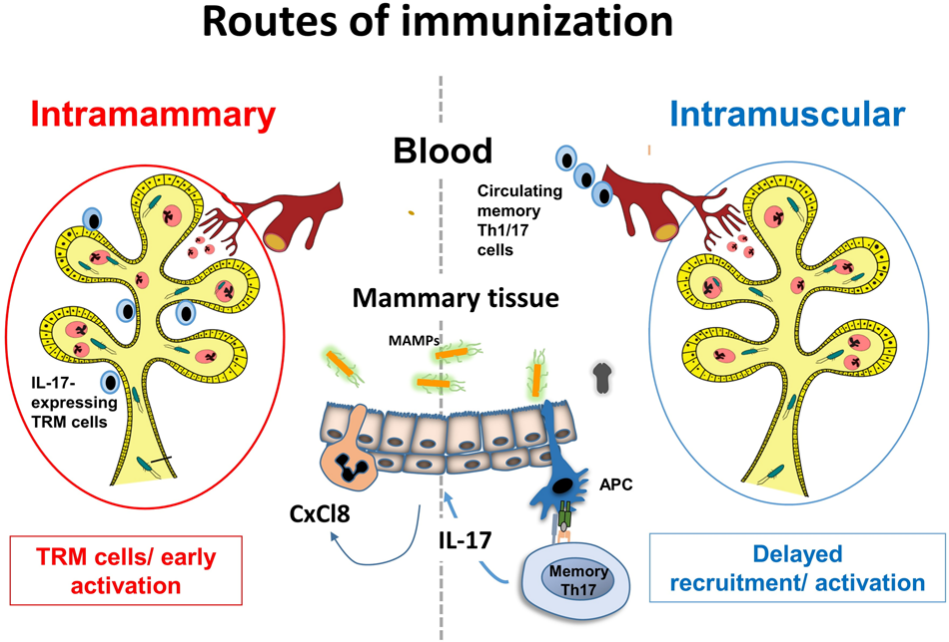
Local mammary immunization establishes resident memory CD4 T cells in the mammary tissue on the contrary to the intramuscular route of immunization. The schematic describes our current view of the beneficial effect of intramammary immunization and compares the two routes of immunization. Systemic injection of killed bacteria in adjuvant produces a pool of Th1/Th17 circulating CD4 T cells. Injection of *E. coli* culture supernate antigens via the teat ductal route enables settlement of CD4 TRM cells in the mammary tissue that are poised to express IL-17 and mobilize a protective response through neutrophil recruitment early during the course of an *E. coli* mastitis.

To summarize we provided some evidence that the two routes of immunization and two different forms of *E.* coli antigens gave different results of immune activation and protective immunity against mammary *E. coli* infection (Fig. 7). The local route of immunization provided some mechanisms of *E. coli* mastitis control above what was observed in adjuvant-only receiving cows. Systemic immunization was helpful to generate a circulating pool of antigen-specific activated/memory T cells that may be mobilized after mammary infection. However, this recruitment occurred later in the course of mastitis and does not seem to be as protective as was the local immunization. Conversely, the antigenic booster enabled the local establishment of TRM cells that are poised to respond from the beginning of the infection. We provide a proof of principle that mammary type 3 immunity may be instrumental in protection against mastitis. Eliciting type 3 immunity in the mammary gland is likely to help the development of new commercial vaccines.

## Methods

### Animals and ethical statement

All experimental procedures and clinical and laboratory records were previously described in Herry et al.^15^. All procedures involving animals were approved by the Ethics Committee of Val de Loire (France), DGRI’s agreement APAFIS#813-2015061109103810v2. Animal studies were compliant with all applicable provisions established by the European directive 2010/63/UE. All methods were applied by approved staff members in accordance with the relevant standard operating procedures approved by the above-mentioned Ethics Committee. All animals used in this study were handled in strict accordance with good clinical practice and all efforts were made to minimize suffering. In brief, 18 Holstein-Frisian heifers in total were synchronized and inseminated at 15 month-old on average. During the last trimester of the gestation, 12 heifers were immunized using IM injections with heat-killed *E. coli* P4 emulsified in Montanide ISA 61VG® (Seppic, France) oil adjuvant, as recommended by the manufacturer. Two months later, half of the heifers (IM group, *n* = 6) got a second injection as a booster. The others received 50 µg of protein concentrate prepared from a *E. coli* P4 supernate in two quarters (IMM group, *n* = 6). Cows of the control group (CONT, *n* = 6) were injected intramuscularly with adjuvant only twice at the same dates. Cows were then challenged on average at 49 days in milk in one healthy quarter by infusion of a *E. coli* P4 bacterial suspension (10^3^ bacteria).

Forty days after the first challenge, a new mammary infusion with *E. coli* P4 was performed using the same conditions as before, in order to collect tissue samples and to assess the local response. Six previously challenged cows (3 CONT, 3 IMM) were inoculated in one healthy (SCC < 50, 000 cells/mL), and not previously challenged quarter. Clinical data were not collected. SCCs were assessed and *E. coli* bacteria were re-isolated in order to check that inoculation was effective at inducing a mastitis. Cattle were euthanized 16 h after challenge and just before tissue harvest.

### CD4 T cell isolation

Mammary tissue specimens from challenged and control quarters were harvested in the parenchyma region. Mononuclear cells were isolated from 2 g of mammary tissue. Briefly, mammary gland homogenates were incubated with collagenase type IV and DNAse in RPMI for 30 min at 37 °C under agitation. Cells were recovered through Percoll gradient centrifugation and stained with PE-labeled CD45 (CC1, Bio-Rad) and FITC-labeled CD4 (CC8, Bio-Rad) mAbs, before cell sorting using a MoFlo Astrios EQ (Beckman Coulter, Brea, CA, USA).

### RNA extraction

Blood samples were collected in EDTA tubes from the jugular vein at several time points, put on ice, and immediately treated with lysis buffer before storage at −80 °C. Total RNA was extracted from these samples with Nucleospin® RNA Blood kit (Macherey-Nagel). The amount of RNA was determined by measuring 260 nm absorbance using a NanoDrop ND-1000 (NanoDrop Technologies, USA). RNA quality was checked by measuring RIN with a Bioanalyzer (Agilent Technologies, USA). Measured values ranged between 7.8 and 9.3. RNA from isolated CD4 T cells was extracted using Nucleopin® XS kit (Macherey-Nagel).

### Library preparation

RNA-seq was performed at the GeT-PlaGe core facility, INRA Toulouse. RNA-seq libraries have been prepared according to Illumina’s protocols using the Illumina TruSeq Stranded mRNA sample prep kit to analyze mRNA. Briefly, mRNA was selected using poly-T beads. Then, RNA was fragmented, and double stranded cDNA were generated before adaptors were ligated. Eleven cycles of PCR were applied to amplify libraries. Library quality was assessed using a Fragment Analyzer and libraries were quantified by QPCR using the Kapa Library Quantification Kit. RNA-seq experiments were performed on an Illumina HiSeq3000 using a paired-end read length of 2 × 150 pb with the Illumina HiSeq3000 sequencing kits.

### RNA-seq data processing and analysis

Read quality was assessed with FastQC software. Reads were aligned in comparison to the bovine reference genome (UMD3.1.87) with STAR software^30^. Count table was generated with Cufflinks/RSEM^31,32^. Identification of differentially expressed genes (DEGs) was performed with DESeq2 package within the R environment (R Core Team (2013)) following standard normalization procedures. False discovery rate was estimated with Benjamini and Hochberg correction. Expression changes were considered significant when both *q* value < 0.01 and a AbsLog2FoldChange > 1, except when otherwise indicated. DEGs were determined with group comparison at each time point. For control group analysis, the design matrix was defined by design = ~time. For group comparison analysis, the design matrix was calculated by design = ~time + group + time/group.

### Determination of cytokine production

Mammary tissue was weighed to normalization before grounding using ceramic beads (Bio-Rad) in a mixture containing Triton X-100 and a anti-proteases cocktail (Sigma). Plasma, milk, and mammary tissue concentrations for 15 cytokines (IL-1α, IL-1β, IL-1RA, IL-2, IL-4, IL-6, IL-17a, IFNγ, CCL-2, CCL-3, CCL-4, CXCL8, CXCL10, and TNFα) were determined using a Custom bovine cytokines MilliPlex xMAP assay (Merck Millipore). Data were recorded on a MAGPIX flow cytometer using Xponent software (Luminex).

### Modular analysis

To identify immune functions activated at each time point of the transcriptome analysis, a modular analysis was performed with tmod package using normalized count table and modules created by Chaussabel et al.^33^ and Li et al.^34^ as previously described.

### Weighted correlation network analysis (WGCNA)

WGCNA was used to identify co-expressed genes associated with mammary and systemic clinical scores. The WGCNA R package was used for analysis^35^. The minimum module size was set to 30, with a merge cut height of 0.25. The soft-thresholding power as a function of the scale-free topology index was defined at six. In this construction, all pairwise comparisons were conducted between control, IM, and IMM groups. Modules with correlation (*r* ≥ 0.5) and *p* value (≤0.05) were extracted for further inquisition. WGCNA module annotation was carried out with ToppFun^36^. A hypergeometric test with Bonferroni correction was applied to select statistically significant terms.

### Data visualization

PCA and volcanoplot were prepared with DESeq2 rlog-normalized RNA-seq data only. For PCA, the top 500 most variable genes were used. The EnhancedVolcano R package was used for volcanoplot^37^ and the pcaExplorer^38^ was used for PCA. The pheatmap package was used for heatmap visualization^39^. The clustering was based on Euclidean distance. Boxplox graphs were drawn using tmod R package^33,34^. To visualize gene expression and networks, Ingenuity Pathways Analysis (IPA, Qiagen) solfware was used to create network and to examine genes functions. Gene set enrichment analysis was represented in a dendrogram created with the IDEP R script^40^. The network analysis was carried out with IPA Software (Qiagen). Barplot and dot plot representation were performed with GraphPad Prism version 6.01 for Windows (GraphPad Software, La Jolla California USA).

### Statistical methods

All data are presented as the mean value ± SD for *n* = 6 cows per experimental group, with individual values showed as a dot. Statistical analyses were performed using R software by applying Kruskal-Wallis, Mann–Whitney, or repeated measures Wilcoxon tests. The statistical significance was considered with a threshold *p* value below 0.05, except otherwise indicated.

### Reporting summary

Further information on experimental design is available in the Nature Research Reporting Summary linked to this paper.

## Supplementary information

Supplementary Information

Reporting Summary Checklist

## Data Availability

Sequence data that support the findings of this study have been deposited in Gene Expression Omnibus (GEO) with the accession number GSE159283 and GSE159286.
