# Supplementary material for: Th17-related mammary immunity, but not a high systemic Th1 immune response is associated with protection against E. coli mastitis
Source: NPJ Vaccines. 2020 Nov 24;5:108. doi: 10.1038/s41541-020-00258-4 (PMC7686320; doi:10.1038/s41541-020-00258-4)
Supplement: Supplementary file 1 — Supplementary Information [file 41541_2020_258_MOESM1_ESM.pdf]

Supplemental data

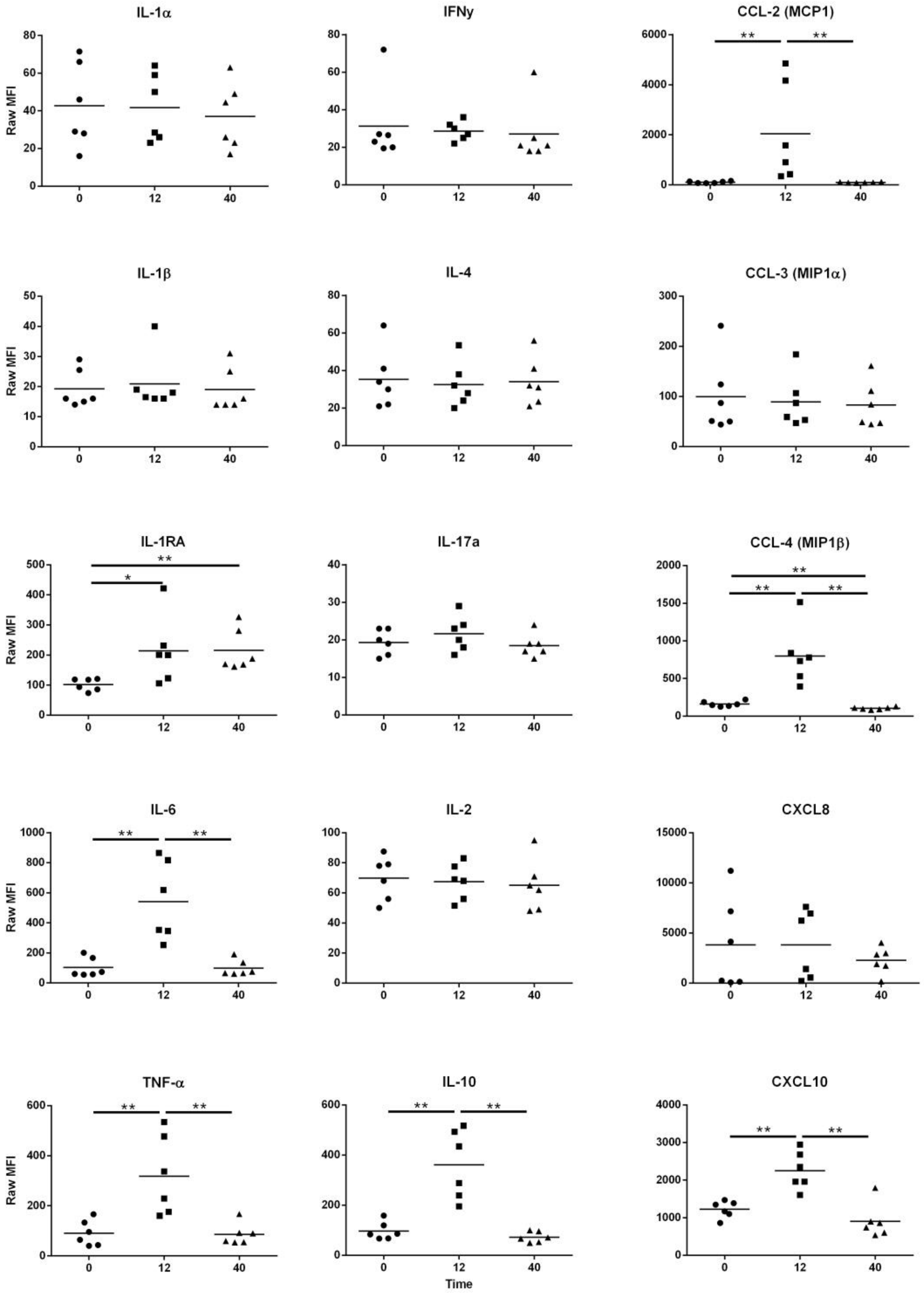

Supplementary Figure 1. **Blood cytokine profile in control cows during the *E. coli* mastitis course at three time points.** Dot plot with mean and individual values of plasma cytokine concentrations of control cows at 0, 12, and 40 hpi are shown. Measures are expressed as raw mean fluorescence intensity (MFI) using a Custom bovine cytokines MilliPlex assay. Statistical analysis was done with Kruskal wallis and only significant differences (* : p<0.05 ; ** : p<0.01 ; *** : p<0.001) are shown.

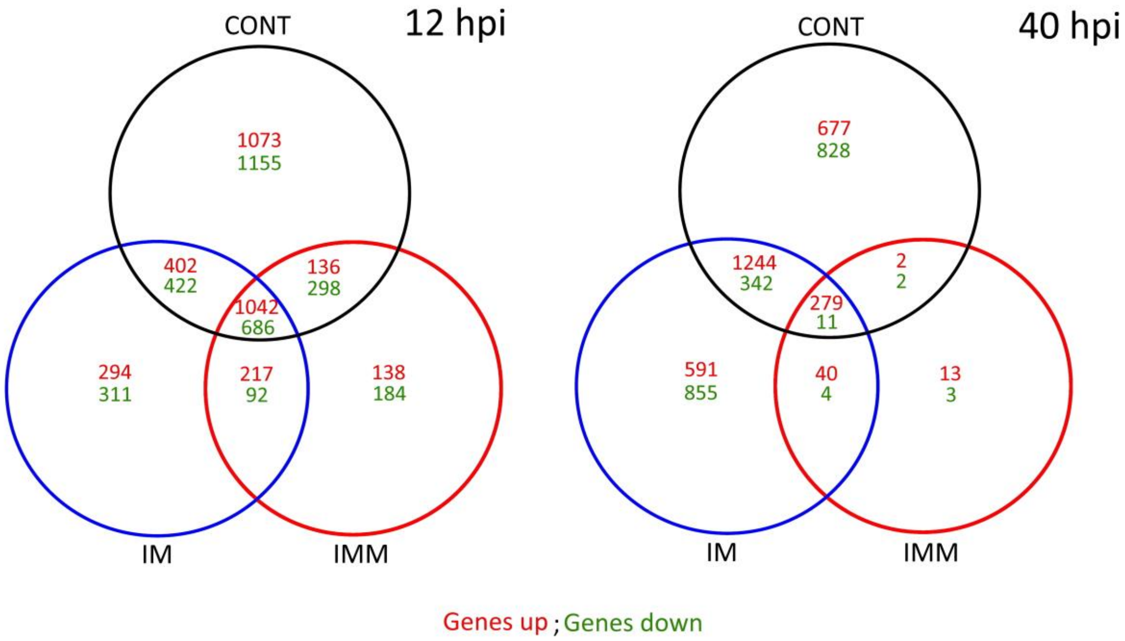

Supplementary Figure 2. Number of differentially-expressed genes amongst control and immunized cows at 12 and 40 hpi compared to expression at 0 hpi during the *E. coli* mastitis course. DEGs between time points were assessed in each group using Deseq2. Venn diagram of identified genes in control, intramuscular- and intramammary-immunized groups at 12 hpi (**a**) at 40 hpi (**b**) are shown with the number of genes and the direction of changes presented between conditions.

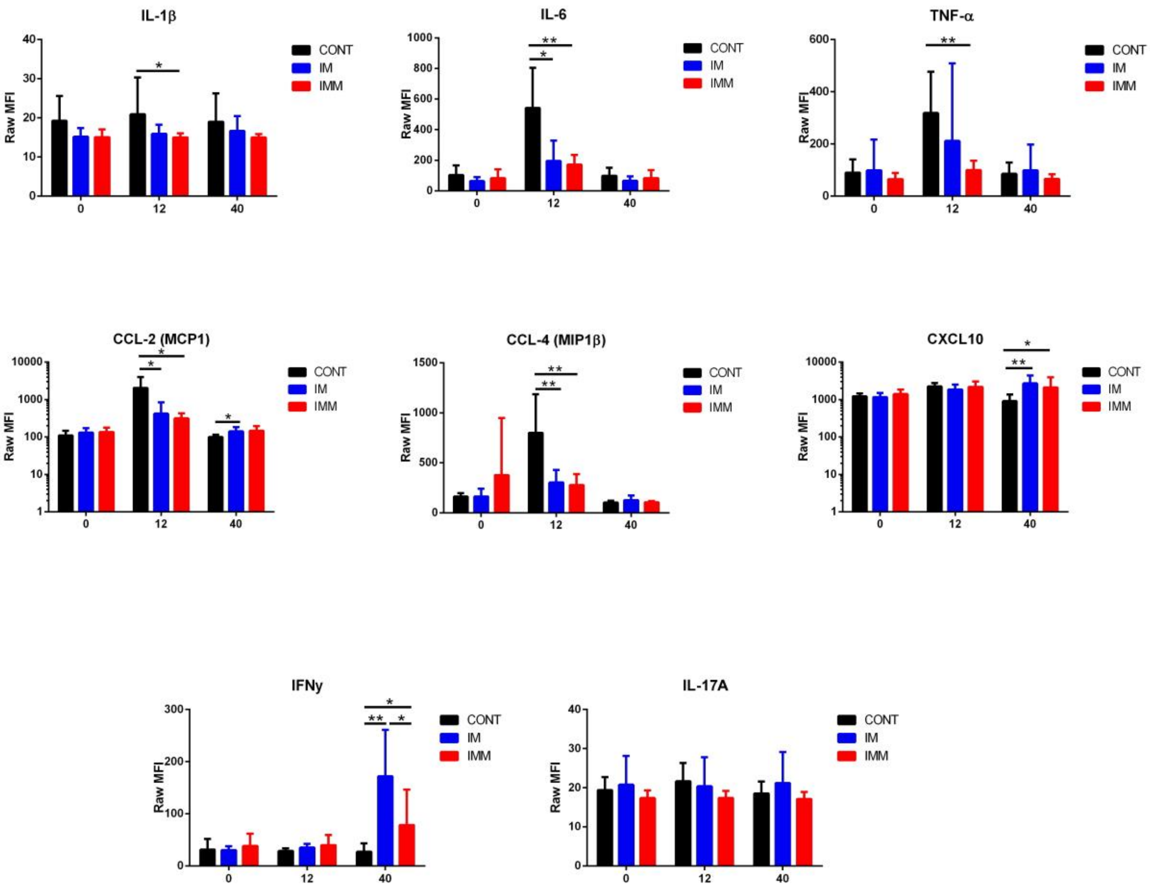

Supplementary Figure 3. Cytokine profiles in plasma of control and immunized cows during the *E. coli* mastitis course. Measures are expressed as raw mean fluorescence intensity (MFI) using a Custom bovine cytokines Milliplex assay and presented as barplots with mean and standard deviation for control and immunized cows at 0, 12, and 40 hpi. Only significant differences are shown after statistical analysis (Krustal wallis (* : p<0.05 ; ** : p<0.01 ; *** : p<0.001)).

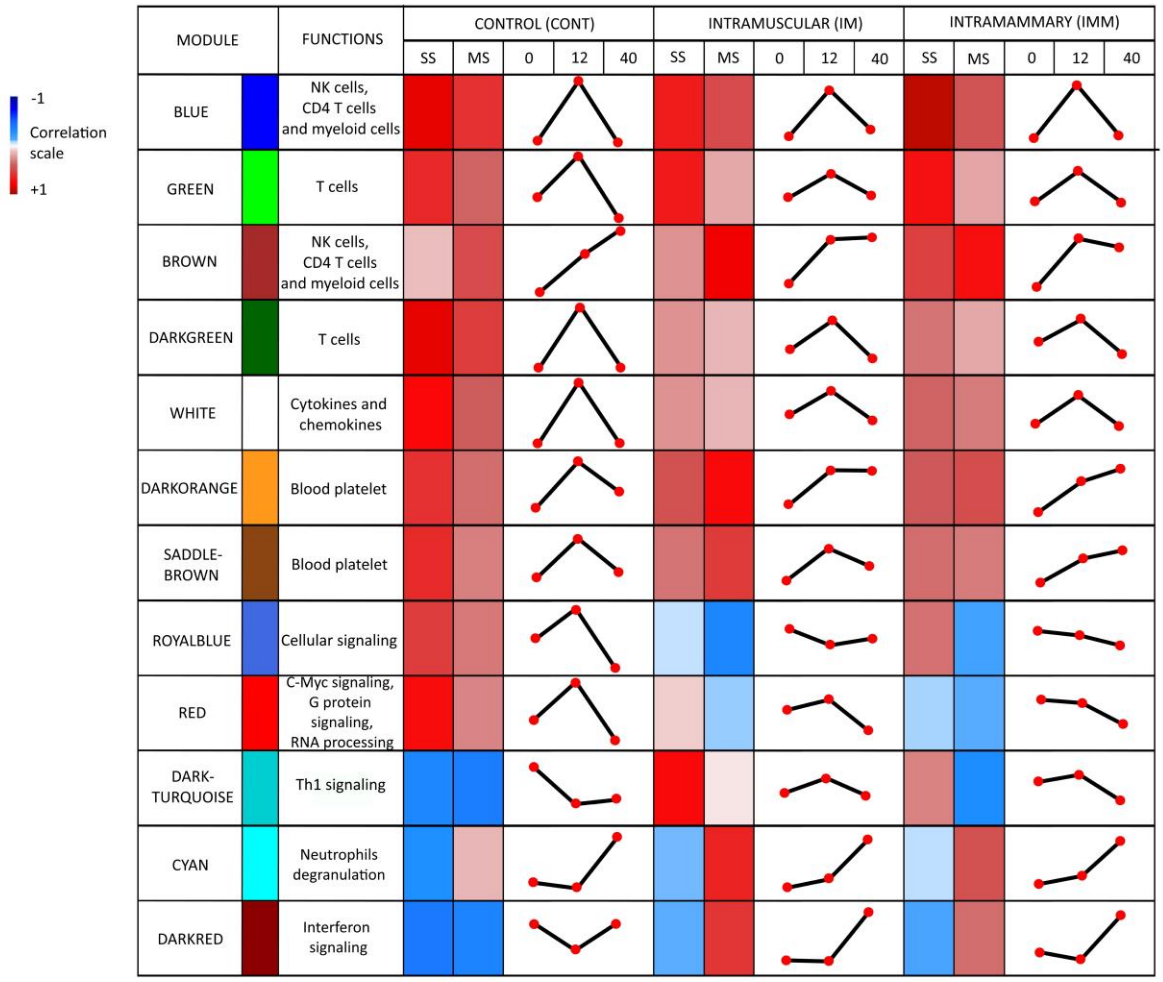

Supplementary Figure 4. Weighted correlation network analysis (WGCNA) of control and immunized cows during *E. coli* mastitis. Only modules with at least 30 genes were retained. Module activation is presented according to the time. Blue and red colors are for modules correlating with the mammary (MS) and the systemic (SS) score, respectively. Functions of each module were determined using the GSEA method.

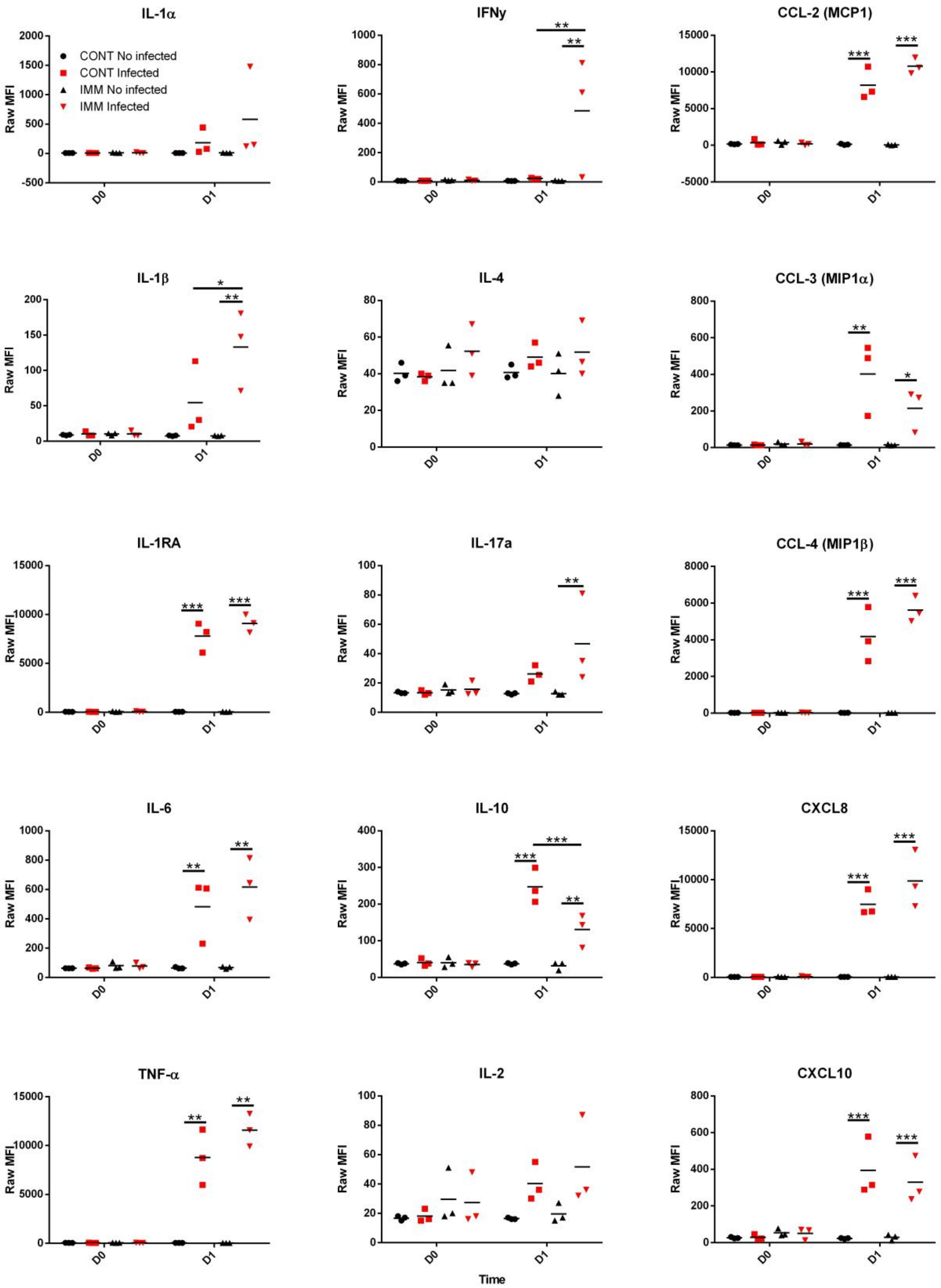

Supplementary Figure 5. Cytokine profile in milk of control and immunized cows during *E. coli* mastitis. Measures are expressed as raw mean fluorescence intensity (MFI) using a Custom bovine cytokines MilliPlex assay and presented as individual values with the mean, for control and immunized groups at 0 and 16 hpi in milk of non-infected and challenged quarters. Statistical analysis was done with Kruskal wallis and only significant differences are shown (* : p<0.05 ; ** : p<0.01 ; *** : p<0.001)

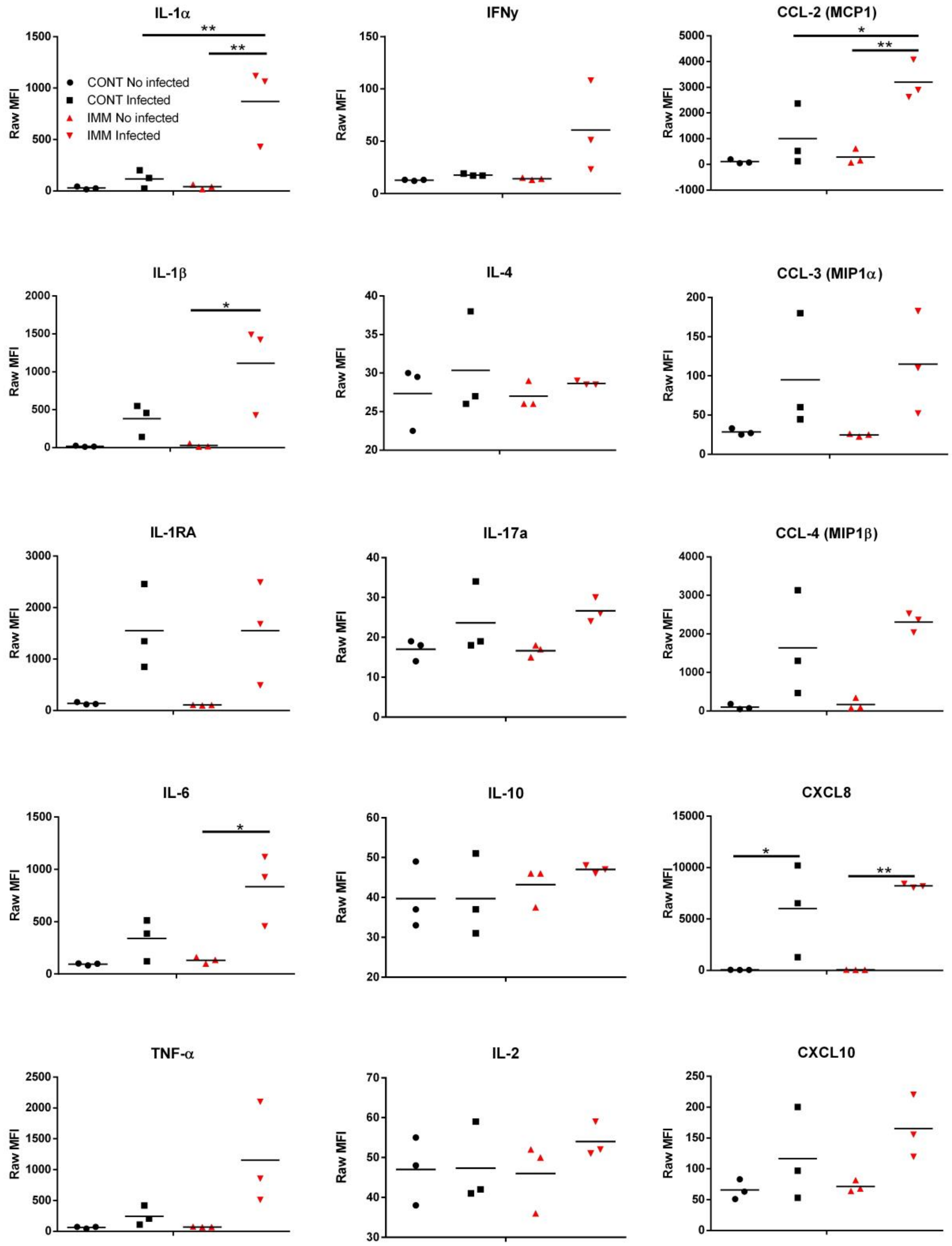

Supplementary Figure 6. Cytokine profile in mammary tissue of control and immunized cows during *E. coli* mastitis. Measures are expressed as raw mean fluorescence intensity (MFI) using a Custom bovine cytokines MilliPlex assay and are presented as individual values with the mean, for control and immunized groups in non-infected and challenged quarters. Statistical analysis was done with Kruskal wallis and only significant differences are shown (* : p<0.05 ; ** : p<0.01 ; *** : p<0.001)

Supplementary Table 1. Reads numbers of each sample at each time (Expression in millions)

animal	group	time	Reads mapped number (x milions)	% mapped reads
274	CONT	0 hpi	5.77113	92.18
275	CONT	0 hpi	9.206225	87.7
279	IM	0 hpi	11.877493	87.8
281	IM	0 hpi	11.210751	93.9
282	CONT	0 hpi	6.700487	83.6
283	IMM	0 hpi	14.652107	91.49
284	IMM	0 hpi	12.326351	93.27
286	IM	0 hpi	12.137065	92.53
289	IM	0 hpi	8.285817	91.7
292	IMM	0 hpi	6.608721	91.6
293	CONT	0 hpi	11.987429	92.7
297	CONT	0 hpi	9.07678	91.6
3725	IMM	0 hpi	6.279465	91.6
3727	IM	0 hpi	11.138122	86.47
3729	CONT	0 hpi	9.444594	94
3732	IM	0 hpi	10.507804	84.94
3734	IMM	0 hpi	9.274266	92.1
3735	IMM	0 hpi	11.806309	87.6
274	CONT	12 hpi	9.627493	88.7
275	CONT	12 hpi	5.42378	91.6
279	IM	12 hpi	11.323308	90.86
281	IM	12 hpi	6.058263	93.22
282	CONT	12 hpi	7.686087	89.61
283	IMM	12 hpi	9.771432	89.59
284	IMM	12 hpi	11.752525	90.13
286	IM	12 hpi	5.391165	93.77
289	IM	12 hpi	10.610801	92.37
292	IMM	12 hpi	11.362103	92.53
297	CONT	12 hpi	9.981453	90.04
3725	IMM	12 hpi	14.206376	93.67
3727	IM	12 hpi	12.614888	89.92
3729	CONT	12 hpi	8.730695	90.49
3732	IM	12 hpi	9.186363	78.02
3734	IMM	12 hpi	10.152582	92.1
3735	IMM	12 hpi	10.334289	92.1
274	CONT	40 hpi	13.612505	94.48
275	CONT	40 hpi	12.064725	90.96
279	IM	40 hpi	14.373269	94.03
281	IM	40 hpi	11.494359	91.16
282	CONT	40 hpi	8.456053	86.88

283	IMM	40 hpi	9.527324	93.9
284	IMM	40 hpi	11.313552	92.1
286	IM	40 hpi	15.885142	90.66
289	IM	40 hpi	9.426813	93.4
292	IMM	40 hpi	6.478535	92.21
293	CONT	40 hpi	9.940772	90.4
297	CONT	40 hpi	10.033062	77.8
3725	IMM	40 hpi	12.385064	93.74
3727	IM	40 hpi	8.493025	93.63
3729	CONT	40 hpi	11.50606	90.1
3732	IM	40 hpi	15.201339	87.6
3734	IMM	40 hpi	12.364367	90.03
3735	IMM	40 hpi	11.176495	87.64
274	CONT	B7	9.38013	93.51
275	CONT	B7	11.122096	90.27
279	IM	B7	7.476581	91.44
283	IMM	B7	13.88473	91.07
284	IMM	B7	6.078106	93.78
286	IM	B7	9.42854	91.61
289	IM	B7	4.85905	92.21
292	IMM	B7	11.15113	94.4
293	CONT	B7	10.791246	91.72
297	CONT	B7	9.519881	80.02
3725	IMM	B7	10.55349	91.08
3727	IM	B7	10.272195	92.31
3729	CONT	B7	12.610108	93.15
3732	IM	B7	9.782585	84.27
3734	IMM	B7	12.731626	92.33
3735	IMM	B7	9.572695	79.85
274	CONT	I0	8.492296	90.81
275	CONT	I0	7.96908	88.23
279	IM	I0	13.671551	87.26
281	IM	I0	12.873443	89.47
282	CONT	I0	10.279045	83.71
283	IMM	I0	13.358223	90.5
284	IMM	I0	11.070205	88.58
286	IM	I0	8.797038	91.73
289	IM	I0	13.204483	89.35
292	IMM	I0	10.715812	85.81
293	CONT	I0	12.608356	92.16
297	CONT	I0	14.406925	92.31
3725	IMM	I0	8.397915	90.78
3727	IM	I0	10.330763	89.39
3729	CONT	I0	15.504844	92.85
3732	IM	I0	11.790845	91.7

3734	IMM	I0	10.390356	91.24
3735	IMM	I0	10.188433	93.34
274	CONT	I7	8.456255	86.06
275	CONT	I7	10.281408	92.33
279	IM	I7	10.228077	89.6
281	IM	I7	10.250527	90.44
282	CONT	I7	13.761794	87.97
283	IMM	I7	12.322651	92.3
284	IMM	I7	13.846079	91.56
286	IM	I7	11.010188	91.99
289	IM	I7	13.940961	92.78
292	IMM	I7	12.354945	90.63
293	CONT	I7	11.328832	92.36
297	CONT	I7	12.745967	93.37
3725	IMM	I7	13.617075	92.38
3727	IM	I7	6.735543	91.42
3729	CONT	I7	12.29389	90.21
3732	IM	I7	7.564474	88.89
3734	IMM	I7	9.17878	91.5
3735	IMM	I7	12.332537	92.87

Supplementary Table 2 Top 10 genes up or down regulated in control group after DESeq2 comparison between 0 hpi and 12 hpi.

T12vsT0 (up)	Gene Symbol	Log2FoldChange	Q-value
1	PTX3	10,66	1,48E-221
2	TNFAIP6	5,51	1,31E-69
3	ALPL	6,97	9,08E-65
4	DNAJB1	3,83	6,02E-58
5	SLCO4A1	4,09	1,27E-56
6	HSPA1A	5,66	1,08E-50
7	HSPH1	4,92	1,74E-46
8	IL1RAP	4,2	1,27E-45
9	IL18R1	4,11	3,53E-45
10	RAB3IP	4,53	6,45E-42
T12vsT0 (down)			
1	GDA	-6,14	2,51E-88
2	CTSB	-3,08	5,90E-77
3	TNFSF13	-4,18	5,03E-60
4	CALCRL	-6,12	2,23E-58
5	CKB	-3,76	5,04E-53
6	GM2A	-3	9,40E-53
7	IGSF8	-2,63	2,82E-51
8	CCR2	-2,78	4,78E-51
9	NLRP1	-5,53	9,24E-46
10	CSF1R	-3,65	4,95E-42

T40vsT0 (up)	Gene Symbol	Log2FoldChange	Q-value
1	SHROOM4	5,25	9,21E-49
2	KBTBD7	3,12	9,21E-49
3	HIST1H1C	3,08	7,95E-47
4	SLC25A37	2,92	3,36E-43
5	FOXRED1	2,44	8,69E-43
6	ALOX5AP	3,44	2,91E-40
7	RYR1	4,31	4,73E-39
8	KREMEN1	3,83	6,42E-38
9	IL2RA	2,42	1,42E-35
10	PTPN5	3,24	4,28E-35
T40vsT0 (down)			
1	DAB2	-3,35	4,84E-11
2	STEAP4	-2,01	4,01E-09
3	TPC3	-2,36	2,46E-05
4	GRIA3	-4,79	2,46E-05
5	CACNA2D1	-4,97	2,51E-05
6	ZFYVE28	-2,68	3,51E-05
7	CDH10	-3,05	9,07E-05
8	CPB2	-2,27	0,0001
9	UNC5A	-2,56	0,0001
10	IGFBP1	-2,84	0,0001

Supplementary Table 3 Top 10 genes up or down modulated in IM group after DESeq2 comparison between 0 hpi and 12 hpi and 0 hpi and 40 hpi

IM T12vsT0 (up)	Gene Symbol	Log2FoldChange	Q-value
1	SERPINB4	9,53	9,55E-45
2	PTX3	10,48	1,53E-40
3	SOCS3	2,58	1,17E-37
4	TCN1	3,59	1,23E-34
5	SHROOM4	4,88	1,71E-29
6	RAB3IP	4,55	8,71E-29
7	RAB20	3,99	1,57E-28
8	MEGF9	3,41	1,61E-28
9	MMP9	4,14	6,80E-28
10	CPNE2	4,72	5,44E-27
IM T12vsT0 (down)			
1	IFT27	-2,45	7,70E-10
2	CDH10	-3,85	1,43E-08
3	PDK4	-2,12	1,91E-08
4	BATF2	-3,21	2,99E-08
5	CD1A	-3,5	4,06E-08
6	USHBP1	-2,31	3,80E-06
7	IGFBP1	-5,19	1,46E-05
8	SIPA1L2	-2,64	1,78E-05
9	SIGLEC1	-2,24	1,94E-05
10	KLHL33	-2,57	2,85E-05

IM T40vsT0 (up)	Gene Symbol	Log2FoldChange	Q-value
1	KBTBD7	2,58	2,27E-61
2	SLC25A37	2,71	2,73E-45
3	TCN1	3,77	1,83E-44
4	APOBEC3Z1	3,43	8,68E-42
5	RSAD2	2,97	6,97E-41
6	SLC28A3	3,84	6,07E-39
7	CD24	2,61	2,38E-38
8	OAS1X	2,44	2,10E-37
9	FOXRED1	2,21	1,39E-35
10	SHROOM4	4,87	6,16E-35
IM T40vsT0 (down)			
1	ZFYVE28	-3	9,72E-22
2	CD209	-3,4	3,84E-11
3	UNC5A	-4,05	9,70E-10
4	ELANE	-2,63	2,60E-07
5	SLC4A3	-2,45	2,58E-05
6	COL9A2	-4,23	0,0001
7	IGFBP1	-3,24	0,0001
8	DAB2	-3,67	0,0001
9	PCDH8	-4,28	0,0002
10	ADAM28	-2,4	0,0003

Supplementary table 4 Top 10 genes up or down modulated in IMM group after DESeq2 comparison between 0 hpi and 12 hpi and 0 hpi and 40 hpi.

IMM T12vsT0 (up)	Gene Symbol	Log2FoldChange	Q-value
1	PTX3	9,25	4,36E-33
2	SOCS3	2,57	5,00E-33
3	SLCO4A1	3,39	1,21E-31
4	MMP9	3,72	2,12E-31
5	SERPINB4	8,49	1,59E-24
6	RAB20	3,64	7,95E-23
7	RAB3IP	3,78	3,00E-21
8	IL18R1	3,68	4,19E-21
9	ETV5	3,02	4,19E-21
10	ALPL	5,53	2,05E-20
IMM T12vsT0 (down)			
1	HES4	-3,57	9,50E-12
2	SERPINE1	-2,42	1,00E-11
3	CD1A	-4	6,51E-11
4	ZNF618	-2,44	4,93E-10
5	CCDC9B	-2,71	1,91E-09
6	PLXND1	-2,17	4,32E-09
7	VNN1	-2,79	4,73E-08
8	IFT27	-2,32	6,55E-08
9	TSPAN33	-2,17	2,19E-07
10	SIGLEC1	-2,89	2,64E-07

IMM T40vsT0 (up)	Gene Symbol	Log2FoldChange	Q-value
1	CATHL6	21,62	7,97E-09
2	SERPINB4	6,67	7,35E-06
3	PTPN5	2,25	2,67E-05
4	CCDC191	2,31	8,86E-05
5	IFI44	2,37	0,0006
6	ALOX5AP	2,2	0,001
7	NOL3	2,23	0,001
8	RSAD2	3,11	0,001
9	MMP9	3	0,001
10	OAS2	3,14	0,001
IMM T40vsT0 (down)			
1	DAB2	-2,62	0,001
2	IGFBP1	-4,001	0,004

Supplementary Table 5 Top 10 functions up regulated at I7 after DESeq2 comparison between immunized groups and the control group.

IMM+IM vs CONT up (I7)	ID	Name	FDR B&H	Genes from Input	Genes in Annotation
1	GO:0046903	secretion	3.596E-22	118	1835
2	GO:0002274	myeloid leukocyte activation	1.23E-21	68	684
3	GO:0002252	immune effector process	1.23E-21	97	1332
4	GO:0002444	myeloid leukocyte mediated immunity	6.017E-20	59	568
5	GO:0002446	neutrophil mediated immunity	8.895E-19	54	506
6	GO:0002263	cell activation involved in immune response	1.549E-18	65	736
7	GO:0045321	leukocyte activation	2.077E-18	92	1386
8	GO:0002275	myeloid cell activation involved in immune response	2.28E-18	56	557
9	GO:0043299	leukocyte degranulation	4.456E-18	55	546
10	GO:0002366	leukocyte activation involved in immune response	4.456E-18	64	732

Supplementary Table 6 Top 5 up functions at B7 after DESeq2 comparison between B7 and I0

Top functions/Pathways	Category	ID	Name	FDR B&H	Genes from Input	Genes in Annotation
**Genes up in IM group**						
1	Pathway	1269311	Interferon Signaling	8.150E-8	22	202
2	Pathway	1269312	Interferon alpha/beta signaling	5.594E-6	12	69
3	Pathway	1269310	Cytokine Signaling in Immune system	1.130E-5	39	763
4	Pathway	1269203	Innate Immune System	2.105E-5	54	1312
5	Pathway	1269259	RIG-I/MDA5 mediated induction of IFN-alpha/beta pathways	2.219E-5	12	84
**Gene up in IMM group**						
1	Pathway	1269203	Innate Immune System	6.395E-6	66	1312
2	Pathway	1457780	Neutrophil degranulation	7.295E-6	35	492
3	Pathway	1270223	O2/CO2 exchange in erythrocytes	1.159E-4	6	13
4	Pathway	99051	Chemokine signaling pathway	2.315E-3	16	182
5	Pathway	1269318	Signaling by Interleukins	3.046E-3	30	531
**Commun Genes up (CONT-IM)**						
1	Pathway	1269340	Hemostasis	2.438E-2	7	640
2	Pathway	1269350	Platelet activation, signaling and aggregation	2.438E-2	5	282
3	Pathway	M8066	IL22 Soluble Receptor Signaling Pathway	3.889E-2	2	16
4	Pathway	83067	Focal adhesion	3.889E-2	4	199
**Commun Genes up (IM-IMM)**						
1	Pathway	1457780	Neutrophil degranulation	1.447E-17	49	492
2	Pathway	1269203	Innate Immune System	4.731E-11	69	1312
3	Pathway	99052	Lysosome	1.036E-4	14	123
4	Pathway	1269948	Iron uptake and transport	7.795E-3	7	44
5	Pathway	MAP03070	MAP03070 Type III secretion system	7.795E-3	5	19

Supplementary Table 7 Top 10 functions up regulated at 40 hpi after DESeq2 comparison between cured and non-cured cows within the IMM immunized group.

IMMc vs IMMnc down (T40)	ID	Name	FDR B&H	Genes from Input	Genes in Annotation
1	GO:0006955	immune response	5.311E-22	77	1572
2	GO:0006952	defense response	5.928E-20	76	1651
3	GO:0045087	innate immune response	7.502E-14	47	846
4	GO:0051707	response to other organism	5.811E-12	48	986
5	GO:0043207	response to external biotic stimulus	5.811E-12	48	986
6	GO:0032101	regulation of response to external stimulus	1.703E-11	48	1014
7	GO:0006954	inflammatory response	1.722E-11	40	711
8	GO:0009607	response to biotic stimulus	2.768E-11	48	1027
9	GO:0031347	regulation of defense response	1.811E-9	40	820
10	GO:0002252	immune effector process	5.878E-9	40	851
